# Parametric-MAA: fast, object-centric avoidance of metal artifacts for intraoperative CBCT

**DOI:** 10.1007/s11548-025-03348-7

**Published:** 2025-04-05

**Authors:** Maximilian Rohleder, Andreas Maier, Bjoern Kreher

**Affiliations:** 1 Pattern Recognition Lab, Friedrich-Alexander-University, Martenstraße 3, 91058 Erlangen, Germany; 2 Siemens Healthineers AG, Siemensstraße 1, 91301 Forchheim, Germany

**Keywords:** Metal artifact avoidance, Cone-beam CT, CT trajectory optimization

## Abstract

**Purpose:**

Metal artifacts remain a persistent issue in intraoperative CBCT imaging. Particularly in orthopedic and trauma applications, these artifacts obstruct clinically relevant areas around the implant, reducing the modality’s clinical value. Metal artifact avoidance (MAA) methods have shown potential to improve image quality through trajectory adjustments, but often fail in clinical practice due to their focus on irrelevant objects and high computational demands. To address these limitations, we introduce the novel parametric metal artifact avoidance (P-MAA) method.

**Methods:**

The P-MAA method first detects keypoints in two scout views using a deep learning model. These keypoints are used to model each clinically relevant object as an ellipsoid, capturing its position, extent, and orientation. We hypothesize that fine details of object shapes are less critical for artifact reduction. Based on these ellipsoidal representations, we devise a computationally efficient metric for scoring view trajectories, enabling fast, CPU-based optimization. A detection model for object localization was trained using both simulated and real data and validated on real clinical cases. The scoring method was benchmarked against a raytracing-based approach.

**Results:**

The trained detection model achieved a mean average recall of 0.78, demonstrating generalizability to unseen clinical cases. The ellipsoid-based scoring method closely approximated results using raytracing and was effective in complex clinical scenarios. Additionally, the ellipsoid method provided a 33-fold increase in speed, without the need for GPU acceleration.

**Conclusion:**

The P-MAA approach provides a feasible solution for metal artifact avoidance in CBCT imaging, enabling fast trajectory optimization while focusing on clinically relevant objects. This method represents a significant step toward practical intraoperative implementation of MAA techniques.

## Introduction

Intraoperative cone-beam CT (CBCT) is widely employed for the validation of implant placement in orthopedic and trauma surgeries. In pedicle screw fixation, precise screw positioning is essential for spinal stability, and CBCT offers crucial intraoperative verification of implant alignment. However, the presence of metallic implants generates severe artifacts that degrade image quality, obscuring vital anatomical structures and significantly limiting the clinical utility of CBCT for accurate assessment.

To address the challenge of metal artifacts in CBCT, various approaches have been explored, including post-processing methods like metal artifact reduction (MAR). Techniques such as FS-MAR [4] are effective in mitigating mild-to-moderate artifacts but often struggle with stronger artifacts, limiting their effectiveness in severe cases. Beyond post-processing, trajectory optimization has been studied as an alternative to directly avoid artifacts during image acquisition. The concept of adapting the source-detector orbit to improve image quality is well-established, with a range of techniques reviewed in [3]. Non-circular orbits have shown effectiveness in reducing general artifacts without prior scene information [2]. In spine surgery, angular adjustments of circular trajectories have significantly improved both image quality and clinical assessability [5].

Wu et al. [9] introduced a method using six scout views to optimize a tilted scanning trajectory, aiming to minimize metal interference. However, this approach often prioritizes auxiliary metal objects, such as surgical towers, over clinically relevant implants, leading to suboptimal trajectory choices [7]. To address this, Rohleder et al. [7] proposed a method that computes local artifact distributions, allowing clinicians to prioritize regions-of-interest. These approaches rely on deep learning-based segmentation of metal and raytracing to score candidate projections, which present challenges for real-time implementation on current C-arm systems.

In this work, we propose a novel metal artifact avoidance (MAA) approach that employs parametric representations of the objects of interest, specifically modeling them as ellipsoidal surrogates. This parametric representation allows for efficient evaluation of candidate trajectories by grading projections based on the ellipsoidal models rather than the detailed voxel-based masks. By focusing the optimization process exclusively on the clinically relevant implants, the proposed method computes optimal scanning trajectories nearly instantaneously, even without GPU acceleration.

We summarize the following contributions:

- We introduce a fast and efficient MAA technique based on ellipsoidal surrogate models, which significantly reduces the computational cost of trajectory optimization while targeting clinically relevant implants.
- We train a deep learning model to detect surgical implants in scout views, evaluating it alongside the subsequent ellipsoid-based orbit optimization method on clinical cases.
- We validate the parametric-MAA approach through a cadaver study, showcasing its potential for practical intraoperative applications in avoiding metal artifacts during CBCT-guided procedures.

**Fig. 1:**
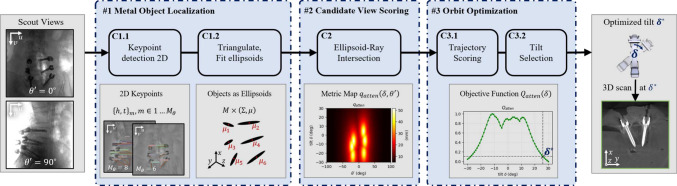
Parametric metal artifact avoidance pipeline. The process begins with two routinely acquired scout views, which are used to detect pedicle screws by identifying the head and tip keypoints (C1.1) and construct an ellipsoidal representation from the triangulated 3D locations of these keypoints (C1.2). Then, candidate projections are scored based on the maximum intersected path length through the ellipsoidal model (C2), and trajectory scores are computed (C3.1) to determine the optimal tilt angle $\delta ^*$ defined as the 95% improvement from the lowest to highest trajectory score (C3.2). The scan performed at $\delta ^*$ is expected to reduce metal bias, thereby minimizing metal artifacts in the final CBCT image

## Materials and methods

The parametric metal artifact avoidance (P-MAA) pipeline shown in Fig. 1 is used to predict the optimal tilt $\delta ^*$ for a given surgical scene. Prior to CBCT acquisition, the intraoperative C-Arm’s isocenter is positioned to the region of interest, typically using a lateral and anterior–posterior view, called scout views. The P-MAA method uses these scout views to first localize metallic objects (step #1), secondly score each view of candidate tilted trajectories (step #2), and finally compute an optimal tilt by minimizing an objective function derived from imaging physics (step #3).

In this study, the P-MAA pipeline is specifically applied to pedicle screws, which are well-suited to representation using ellipsoids due to their elongated, nail-like structure. While this approach is optimized for spinal surgery involving pedicle screws, the choice of shape model may require adaptation for other surgical scenarios involving non-elongated or irregularly shaped metal objects, such as plates, bolts, or pacemakers.

### Localization of metal objects from scout views

The first step in the P-MAA pipeline is detecting metal objects and identifying keypoints, specifically the head and tip of pedicle screws, in scout X-ray images. We use the faster R-CNN architecture, which integrates a region proposal network (RPN) to generate object candidates and a classifier to refine bounding boxes and predict keypoint locations, as commonly applied in tasks like COCO keypoint detection [6].

Preprocessing includes clipping the raw X-ray images to the median plus three standard deviations, converting them to line integrals via a logarithmic transform, and subtracting a low-pass filtered version to handle intensity gradients. The images are then converted to RGB format for compatibility with the Detectron2 framework [10]. After processing, we obtain 2D bounding boxes and keypoint locations for each detected object.

To optimize imaging trajectories, we represent metal objects with ellipsoids, which compactly capture their position, orientation, and extent. Using calibrated projection matrices, the keypoints from the scout views are back-projected into 3D space, generating rays for triangulation. Two object proposals are matched when the head and tip rays intersect within 5 mm each. The closest midpoint between intersecting rays is defined as the 3D head or tip coordinate. Ellipsoids are fitted to these 3D points, aligning their principal axis with the head-to-tip direction and scaling it by the head-tip distance, while the second and third axes are set to 5 mm to represent common screw diameters. Each ellipsoid is represented by its centroid $\mu _m {\in \mathbb {R}^{3}}$ and covariance matrix $\Sigma _m {\in \mathbb {R}^{3 \times 3}}$, forming the set of ellipsoids:

1 $$\begin{aligned} \mathcal {E} = \{ \mathcal {E}_m \mid \mathcal {E}_m = \left( \mu _m, \Sigma _m \right) , \, m = 1, 2, \ldots , M \} \end{aligned}$$

These ellipsoids are used in the subsequent trajectory optimization step.

**Table 1:** Dataset distribution and splits

	Simulated	Cadaver	Clinical	$\sum$	Total images
Train	129	10	0	139	27.800
Validation	9	2	0	11	2.200
Test	18	5	10	33	6.600
Total	156	17	10	183	36.600

Each 3D scan provides 200 images, corresponding to an inter-view angle of 1 degree between consecutive images

#### Model training and data

The faster R-CNN model with a ResNet50 backbone was implemented and trained using the Detectron2 framework [10]. Training was conducted from scratch over 100,000 iterations (approximately 60 epochs), using a learning rate of $25\times 10^{-5}$ with scheduled decays at 60% and 80% of the total training steps. The model was configured with a region proposal network (RPN) generating 256 proposals per image, and the maximum number of detections per image was set to 20. Data augmentation strategies, including random rotation, scaling, and horizontal flipping, were employed to improve generalization and mitigate overfitting. Model training was conducted on an Nvidia Quadro RTX 5000 and took approximately five days. The checkpoint with the lowest validation loss was selected for evaluation. For details on the faster R-CNN architecture, we refer the reader to the original paper [6].

The training and evaluation data consisted of both simulated and real 3D scans. Simulated data were generated from 56 cone-beam CT (CBCT) volumes without metallic objects. Pedicle screw meshes, ranging in length from 35 to 65 mm and diameter from 4 to 7.5 mm, were provided by Nuvasive, San Diego, California. The screws were randomly placed within each volume. Projection images were simulated using a poly-energetic forward model that accounted for physical effects like noise and scatter. Beam hardening effects were inherently incorporated through the use of polychromatic X-ray spectra, while photon starvation was implicitly modeled by the combination of scatter, noise, and reduced primary signal in low-intensity regions. These digitally reconstructed radiographs (DRRs) were generated using the DeepDrr framework [8]. For each scene, 200 projection views were created along a 200-degree arc, using projection geometry similar to the Siemens Cios Spin CBCT system.

The real data comprised 14 cadaver scans and 10 clinical scans. The cadaver scans included instrumentation of the cervical, thoracic, and lumbar spine with pedicle screws. The clinical scans, obtained from the VITOS Clinic in Kassel, Germany, featured real-world surgical scenarios with additional tools and lower image quality. Manual labeling was used to annotate the screws in 3D, which were then projected onto 2D images using the DICOM projection matrices.

The dataset was divided into training, validation, and test sets, as summarized in Table 1. For each 3D scene, 200 projection images were selected, resulting in a total dataset size of 36,600 images, with contributions from simulated, cadaver, and clinical data. Note that we use the entire stack of images from each 3D scan for training, while during application, only one anterior–posterior and lateral scout views will be used.

### Trajectory optimization from ellipsoidal shape models

With the objects in the scene modeled as ellipsoids, component C2 in Fig. 1 aims to estimate the contribution of each view to the metal artifacts that arise during image reconstruction. Metal artifacts, including streaking and blooming artifacts, occur due to the disparity between the idealized assumptions in the reconstruction algorithms and the complex physical processes involved in X-ray imaging. Notable sources of these artifacts include beam hardening, scatter, and photon starvation, among others [1].

In previous studies, several predictors of metal artifact strength have been proposed, including spectral shift, scatter-to-total ratio, and total attenuation, which model the absorption strength in proportion to the radiated metal length [7, 9]. In this work, we focus on total attenuation, as it is a simple and effective predictor of metal artifacts, especially those caused by photon starvation, which is critical in pedicle screw surgeries. The phenomenon of screw shaft blooming, where the screws appear larger than their actual size, can severely impact the clinical utility of CBCT for verifying screw placement in the pedicle channel.

We adapt the total attenuation formulation, $q_\textrm{atten}(\theta ', \delta )$, from [9], which predicts the degree of metal artifact by assessing the maximum intersection of X-rays with the metallic objects, here ellipsoids $\mathcal {E}_m$, in each C-arm pose $(\theta ', \delta )$. This is expressed as

2 $$\begin{aligned} q_{\text {atten}}(\theta ', \delta ) = \max _{u,v,\mathcal {E}_m} p(u,v, \mathcal {E}_m \mid \theta ', \delta ), \end{aligned}$$

where *p* is the projected path length image of ellipsoid $\mathcal {E}_m$ in C-Arm pose $\theta ', \delta$. The key insight is that the maximum projection of an ellipsoid is always through its centroid, allowing for efficient computation of $q_\textrm{atten}$ without the need for rendering intermediate path length images $p(u,v|\theta ',\delta )$. As a result, the number of computations required is proportional only to the number of ellipsoids, making this approach significantly faster. While this technique disregards potential ellipsoid overlaps, we hypothesize the impact is minimal, as pedicle screws’ major axes are typically well separated. Any minor axis overlap contributes less to photon starvation in typical screw dimensions, as their diameters are far smaller than their lengths.

Once the metric maps $q_{\text {atten}}$ are computed, they represent the expected contribution of each view to metal artifacts. The goal of trajectory optimization (C3.1 and C3.2) is to select a C-arm tilt that minimizes the metal artifact contribution while remaining practical for clinical use. Although non-circular trajectories have been shown to offer greater potential improvements in image quality [9], we focus on tilted circular orbits to prioritize clinical applicability, as non-circular orbits pose significant regulatory and logistical challenges.

The optimization task, therefore, simplifies to selecting a tilt angle, $\delta ^*$, that minimizes the metal bias along a circular orbit. Mathematically, this can be expressed as finding the tilt that minimizes the maximum attenuation along the orbit:

3 $$\begin{aligned} \delta ^{*} = \mathop {\mathrm {\arg \!\min }}\limits _{\delta } Q_{\text {atten}}(\delta ) = \max _{\theta '} q_{\text {atten}}(\theta ', \delta ) \end{aligned}$$

In practice, large tilt angles can introduce complications, such as the risk of collisions with the operating table and reduced geometric calibration accuracy. Therefore, the optimal tilt, $\delta ^*$, is not necessarily the one that minimizes metal bias entirely but rather the one that balances artifact reduction with practical constraints. In this work, we select $\delta ^*$ semi-heuristically to provide 95% of the maximum possible reduction in $Q_{\text {atten}}$, ensuring sufficient artifact reduction without compromising clinical usability.

### Experimental studies

#### Study 1: Evaluation of detection model performance

In this study, the performance of the trained faster R-CNN detection model is evaluated on the test set as defined in Table 1. The model, which was initially trained on a combination of simulated and cadaver data, is applied to X-ray images from clinical CBCT scans. The ground-truth pedicle screw head and tip locations for cadaver and clinical data are annotated in the reconstructed 3D CBCT volumes by trained experts and re-projected into the X-ray images. The precision of the keypoint is visually verified. Performance is quantified using standard detection metrics, including average precision and recall for different assignment thresholds, alongside keypoint error (KPE) (Euclidean distance between predicted and ground-truth keypoints). The study aims to validate whether the model’s performance in controlled, simulated environments can be generalized to real-world clinical scenarios, where greater variability in image quality and anatomical complexity may impact detection accuracy.

**Table 2:** Performance metrics for pedicle screw detection on the test set

Metric	Clinical		Cadaver		Simulation	
	BBox	Keypoints	BBox	Keypoints	BBox	Keypoints
AP@0.50:0.95	0.660	0.721	0.802	0.903	0.958	0.900
AP@0.50	0.941	0.791	0.982	0.922	0.990	0.931
AP@0.75	0.762	0.751	0.927	0.912	0.990	0.900
AR@0.50:0.95	0.696	0.780	0.843	0.936	0.975	0.935
AR@0.50	0.936	0.846	0.996	0.956	0.999	0.953
AR@0.75	0.782	0.804	0.949	0.945	0.998	0.935

Keypoint error (KPE) [mm]						
Head KPE (mean)	1.95 ± 2.42		0.95 ± 1.44		0.59 ± 0.62	
Head KPE (median)	1.48 ± 1.39		0.59 ± 0.69		0.37 ± 0.66	
Tip KPE (mean)	1.41 ± 1.71		1.33 ± 1.14		0.99 ± 1.93	
Tip KPE (median)	1.04 ± 1.21		1.11 ± 1.15		0.56 ± 0.68	

Results include average precision (AP) and average recall (AR) at different IoU thresholds for both bounding box (BBox) and keypoint detection. Additionally, keypoint error (KPE) for screw head and tip positions is reported, presented as mean and median values with standard deviations

#### Study 2: Comparison of ellipsoid-based vs. segmentation-based view scoring methods

In this study, we compare the ellipsoid-based view scoring method with a raytracing-based approach based on 3D voxelized masks as the description of metal. The goal is to evaluate whether the optimized tilt angles from the ellipsoid method align with those from the more detailed metal masks, and to assess the computational efficiency of both methods.

Two test cases with 6 and 8 pedicle screws are used. The reconstructed CBCT volumes are processed through a metal segmentation network to produce binary voxel masks. Although simple thresholding may suffice for segmenting metal in artifact-free images, we utilized a pre-trained model to enhance the fidelity of the extracted metal masks. Furthermore, the segmentation results were manually refined to ensure a reliable ground truth. Combined with the raytracing-based artifact prediction approach detailed above, the resulting metric maps can be considered ground truth for this study. To prevent error propagation, ellipsoids are fitted to manually labeled head and tip points. Metric maps $q_{\text {atten}}(\theta ', \delta )$ are calculated using both methods.

The objective function $Q_{\text {atten}}(\delta )$ is then computed for each approach, and the optimal tilt angles are compared. Efficiency is assessed by comparing runtimes for metric map generation at different angular resolutions, demonstrating the faster computation of the ellipsoid-based method.

#### Study 3: Impact on metal artifacts—cadaver study

This study assesses the effectiveness of the P-MAA method in predicting and reducing metal artifacts during CBCT imaging in a cadaver setting. Pedicle screws of varying thickness are instrumented in the lumbar spine (L1–L4), and two scans are performed: one with a 0-degree tilt (standard procedure) and another using the optimal tilt predicted by the P-MAA pipeline. Ground truth for artifact severity is derived by comparing reconstructed screw thickness against manufacturer specifications. Additional scans over a tilt range of $\delta \in [-15^\circ , 15^\circ ]$ are used to compare predicted artifact strength with measured blooming artifacts, quantified by the screw thickness. The objective function $Q_{\text {atten}}(\delta )$ is computed for all screws collectively and individually, allowing an analysis of each screw’s contribution to overall artifact severity.

## Results

### Study 1: Generalization of the detection model on clinical data

The quantitative results in Table 2 highlight several key aspects of the model’s performance across clinical, cadaver, and simulated datasets. Notably, the model achieves the highest performance on the simulated data, with an AP@0.50:0.95 of 0.958 for bounding boxes (BBox) and 0.900 for keypoints. This reflects the advantage of controlled environments in simulated data, where noise and variability are minimal.

In contrast, the performance on clinical data is lower, with an AP@0.50:0.95 of 0.660 for BBox and 0.721 for keypoints, indicating the challenges of real-world variability, including additional surgical tools and lower image quality. Despite this, the model still demonstrates robust performance on clinical data, especially with a high AP@0.50 of 0.941 for BBox detection.

The keypoint error (KPE) is another important factor, with a mean KPE of 1.95 mm for the screw head in clinical data, compared to 0.95 mm for cadaver and 0.59 mm for simulated data. The median KPE follows a similar trend, highlighting the increase in localization error in more challenging, real-world data. However, the detection model still achieves reasonable accuracy, especially when considering the increased complexity of clinical environments.

**Fig. 2:**
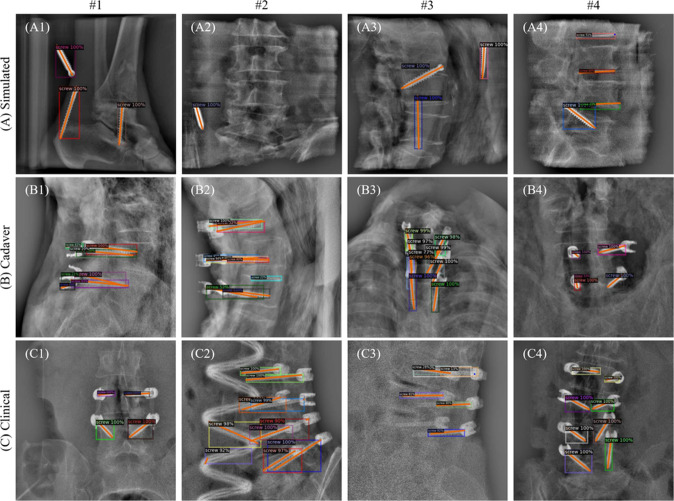
Qualitative detection results. Test set examples from different data sources: **A** Simulated, **B** cadaver, and **C** clinical, showing pedicle screw keypoint detection by the Faster R-CNN model

The examples in Fig. 2 highlight key challenges and successes in the model’s performance across different data sources. In clinical images like C3, high noise in strongly attenuated areas led to a missed screw, while C2 shows that the presence of a surgical tube impeded detection. In contrast, the simulated examples (A1–A4) lack tulip-shaped screw heads, which are present in cadaver and clinical images. Cadaver images (B1–B4) include tulips and have better contrast, with fewer distractions compared to clinical data. Notably, examples like C4 and C1 show the model generalizing well to complex clinical cases. Overall, these results emphasize the model’s adaptability, though performance drops in more complex scenarios.

### Study 2: Comparison of ellipsoid-based vs. segmentation-based view scoring methods

The results from Fig. 4 demonstrate that metric maps $q_{\text {atten}}$ derived from ellipsoids closely resemble those generated from 3D metal masks. While the analytically derived maps show slightly shorter maximal path lengths (50 mm) compared to the raytracing-based ones (60 mm), this difference can be attributed to the ellipsoid models not capturing the tulip shape on top of the screws. Despite this, the objective functions show good alignment, with both methods identifying maxima at nearly identical positions, indicating agreement in the prediction of optimal tilt angles.

**Fig. 3:**
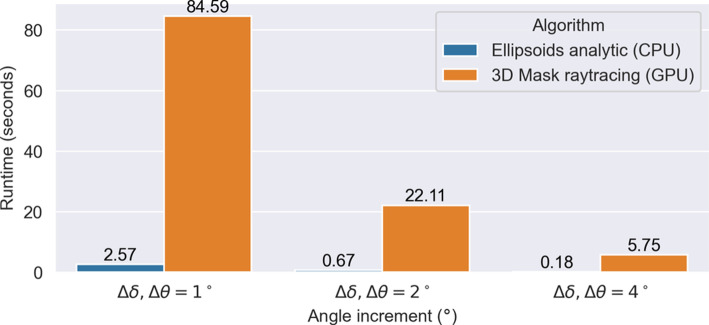
Runtime comparison for three different metric map resolutions. The angle increment between views within a trajectory ($\Delta \theta$) and the increment between tilted trajectories ($\Delta \delta$) is evaluated for the ranges $\delta \in [-30^\circ ,30^\circ ]$ and $\theta \in [-100^\circ ,100^\circ ]$. The pixel size of intermediate rendered images and voxel size of the metal mask were set to 1mm and the number of objects was six

The optimal tilts selected by both methods, using the 95% improvement heuristic, are comparable. However, the ellipsoid-based method tends to be slightly more conservative in its tilt angle recommendations. Importantly, the ellipsoid-based method offers a significant computational advantage, being approximately 33 times faster than the segmentation-based approach. As shown in Fig. 3, this speedup is consistent across different angular resolutions, making the ellipsoid-based method more practical for real-time applications without sacrificing accuracy.

**Fig. 4:**
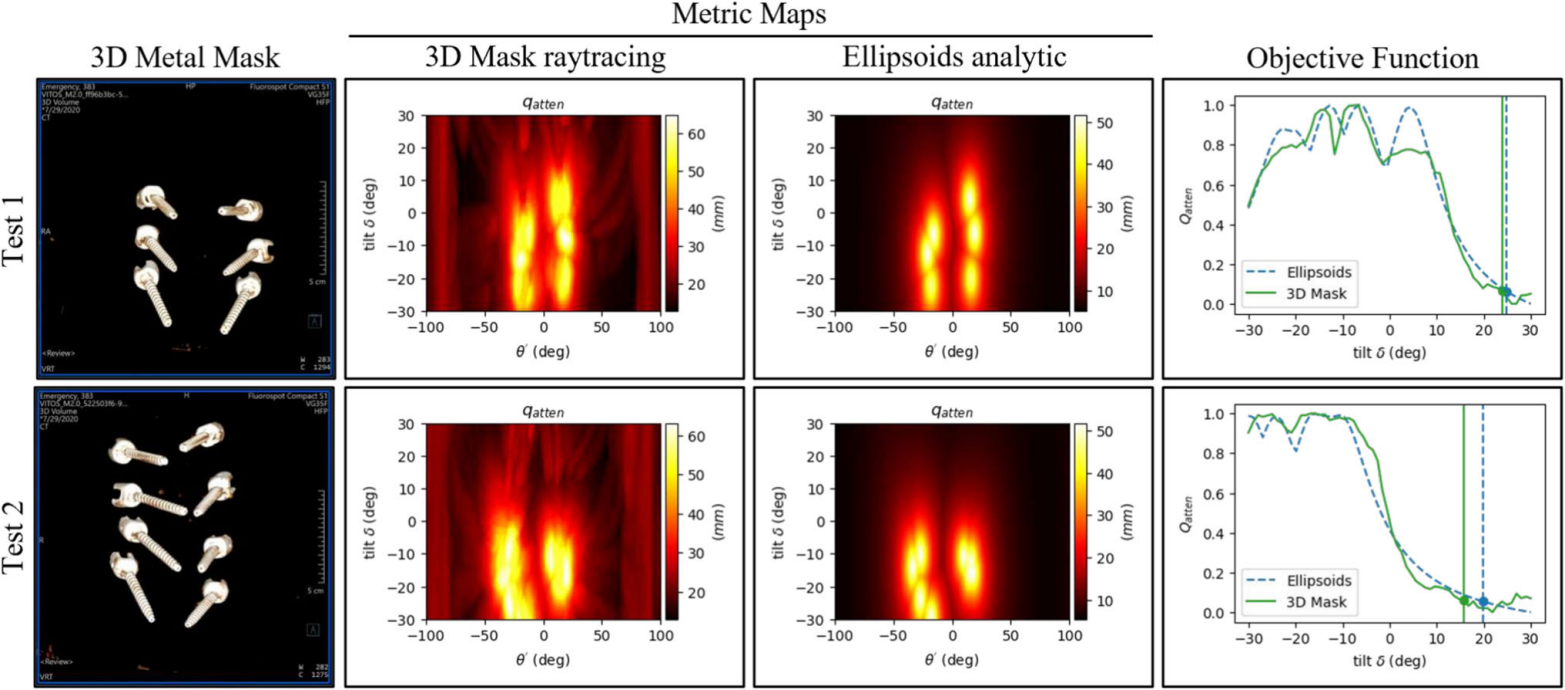
Comparison between ellipsoid and 3D mask-based approach. The metric maps and 1D objective functions derived from ellipsoids and ground-truth metal masks for two samples selected from the test set are shown. The vertical lines show the predicted optimal tilt $\delta ^*$ from the ellipsoid-based method (dashed) and the mask method (solid)

### Study 3: Impact on metal artifacts: cadaver study

This study assesses the effectiveness of the proposed method in reducing artifacts, focusing on the contribution of individual screws to the objective function $Q_{\text {atten}}$.

**Fig. 5:**
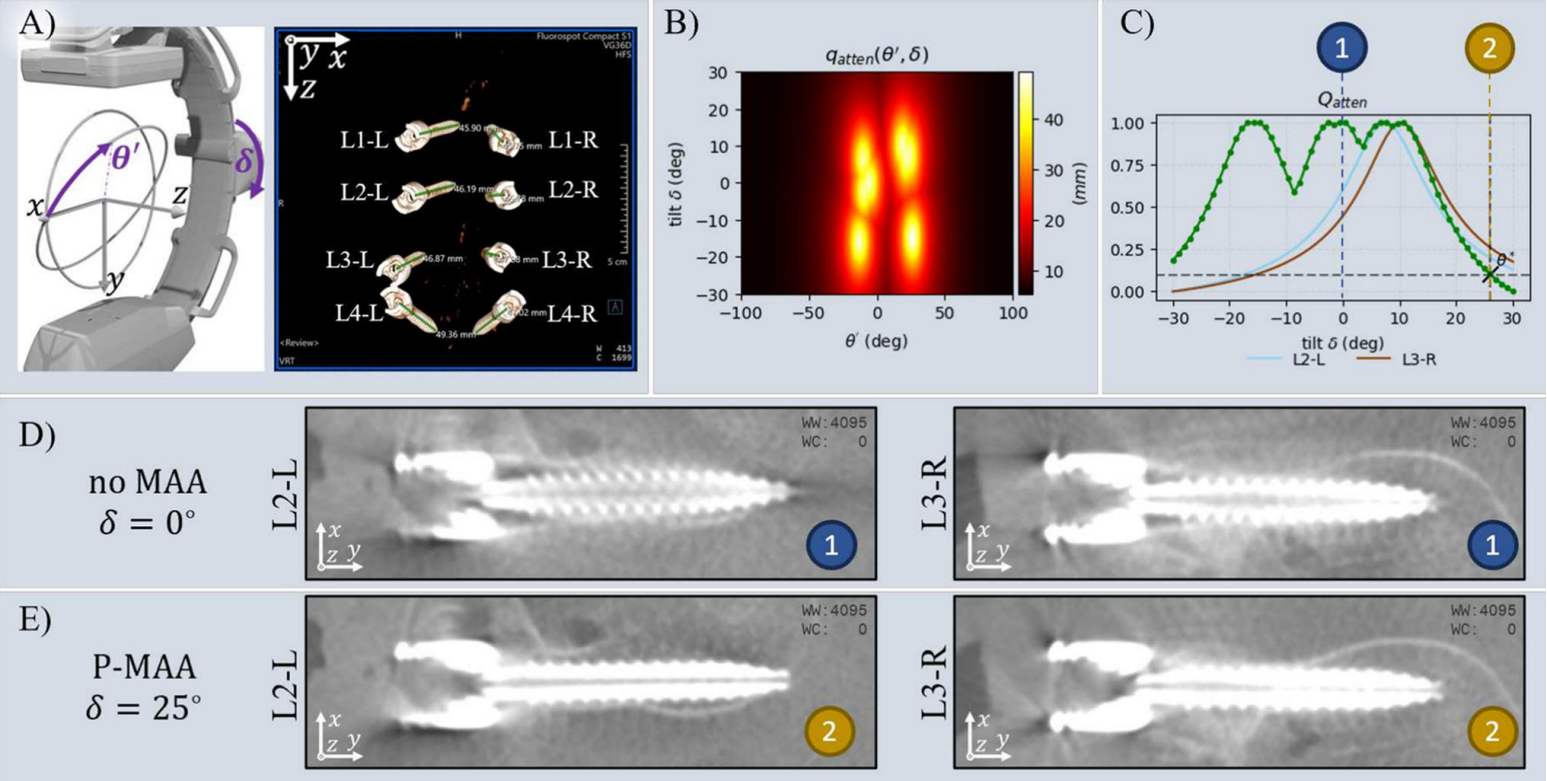
Qualitative results from Study 3—Artifact reduction. A cadaver is instrumented with eight pedicle screws in lumbar spine vertebrae L1–L4, as shown in (**A**). The P-MAA pipeline computes an optimal tilt angle of $\delta ^* = 25^\circ$, illustrated in (**B**) and (**C**). The impact on image quality is demonstrated by comparing the standard trajectory (**D**) to the optimized tilted trajectory (**E**), specifically focusing on screws L2-L and L3-R. The comparison highlights the reduction in metal artifacts achieved using the optimal tilt angle suggested by the P-MAA method

As shown in Fig. 5, the P-MAA pipeline computed an optimal tilt angle of $\delta ^* = 25^\circ$, significantly reducing metal artifacts compared to the standard trajectory. Screws L2-L and L3-R show clearer contours and fewer streaking artifacts, demonstrating the benefit of optimized C-arm tilt.

**Fig. 6:**
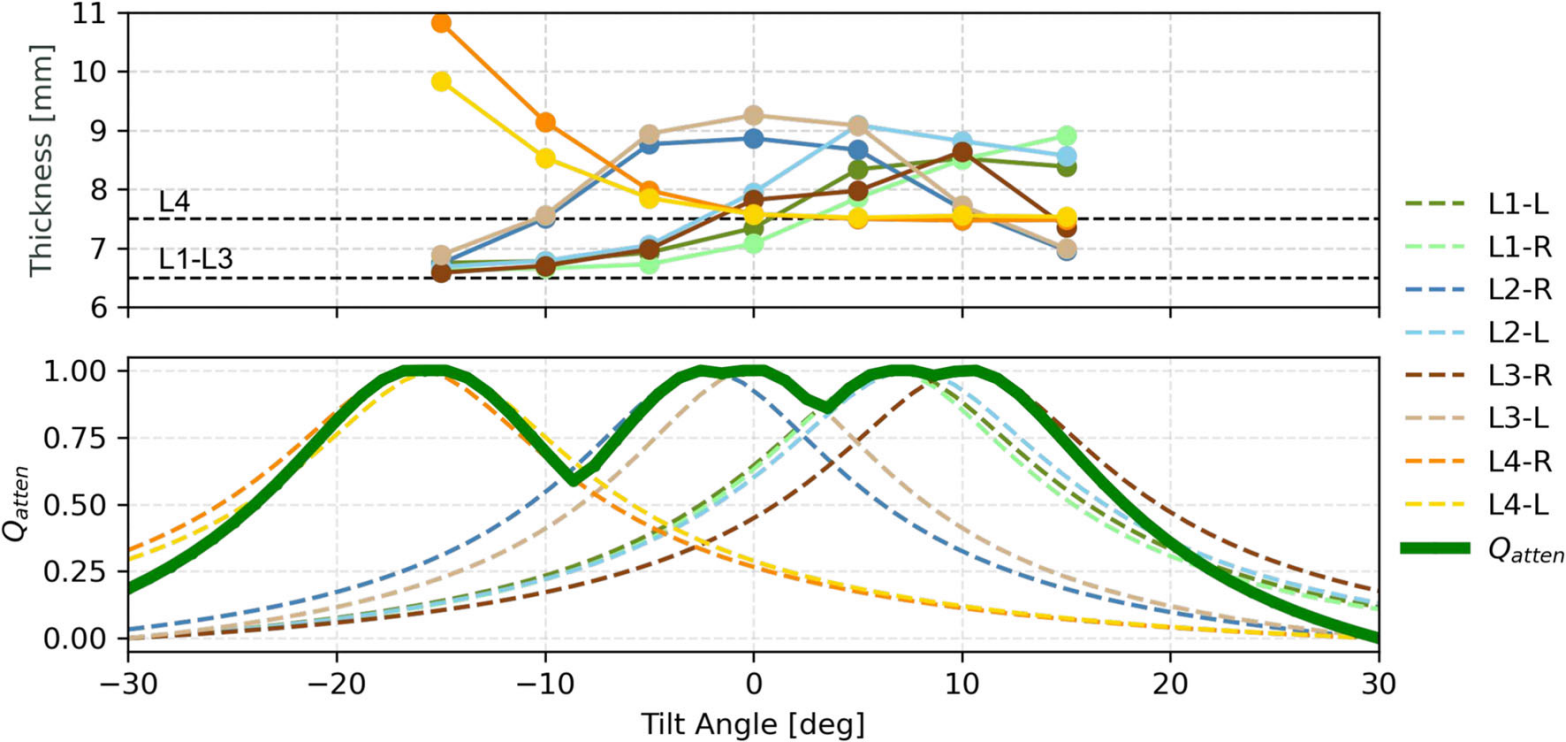
Quantification of blooming artifacts per screw. The top panel shows the measured screw thickness, indicating the severity of blooming artifacts, while the bottom panel compares the predicted artifact severity for each screw, as expressed by the $Q_{\text {atten}}$ objective function. The objective is computed both for all screws collectively and for each screw individually, allowing for direct comparison between measured artifacts and their predicted contribution to $Q_{\text {atten}}$

Figure 6 quantifies these improvements. The top panel shows the measured screw thickness, while the bottom panel compares this to the predicted artifact severity for each screw using the $Q_{\text {atten}}$ objective function. The predictions closely align with the measured blooming artifacts, highlighting the effectiveness of $Q_{\text {atten}}$ in capturing individual screw contributions.

## Discussion and conclusion

The results of this study demonstrate that the detection model trained on a combination of simulated and cadaver data generalizes effectively to clinical scenarios, despite some expected drop in performance. Specifically, the model achieved an AR@0.50 of 0.94 on clinical data, successfully detecting 94% of the screws. This indicates that while there is a performance gap between simulated and real data, the model remains robust in practical applications, especially given the variability in clinical settings.

The proposed method showed clear effectiveness in reducing metal artifacts, as evidenced by the reduction in blooming artifacts and improved image quality in the cadaver study. The object-aware objective function was able to predict blooming artifacts on a per-screw basis, providing a more targeted approach to artifact avoidance. This is particularly important, as it allows for individual screw analysis, which is a unique advantage over segmentation-based approaches, where the notion of discrete objects is less clear.

A significant advantage of the P-MAA method is its computational efficiency. The computation of metric maps from ellipsoidal approximations was shown to be 33 times faster than those derived from detailed 3D metal masks, while maintaining a high level of agreement in predicting artifact severity. However, we assumed that overlap between objects would not have a significant impact on the results. While the metric demonstrated good prediction of blooming artifacts, further research is needed to validate the hypothesis that non-modeled objects, such as surgical towers, do not interfere with the predictions.

One important implication of this work is the potential to expand the application of P-MAA beyond the current focus on pedicle screws. Although this study focused on screws, the general approach could be applied to other metallic objects commonly encountered in surgical settings. The ellipsoidal model, well-suited for elongated objects like screws, may be expanded to model more complex shapes, such as plates or cylinders, through collections of ellipsoids or other geometric primitives.

However, this study has several limitations. First, triangulation from two views is reliable only when keypoints are view-invariant, i.e., unambiguously identifiable from different perspectives. Additionally, when multiple screws lie within the same epipolar plane, the 5-mm tolerance used in this study may lead to correspondence issues. A potential solution for future work would be to incorporate learning-based correspondence matching, which could utilize visual features to solve ambiguous assignment. Furthermore, the entire model pipeline was not tested end-to-end, meaning we did not assess how missed detections may influence the proposed tilts. Additionally, the current method only considered tilted circular orbits, but incorporating additional dimensions of C-arm movement, such as swiveling around the horizontal axis, as suggested by [5], would be a valuable extension. P-MAA’s computational efficiency would make such optimizations feasible within clinically acceptable time frames.

Looking forward, incorporating more realistic clinical data into the training set would likely improve detection rates and further close the gap between simulated and real-world performance. Future developments in object-centric modeling could also include task-specific optimizations, which could resolve conflicts in orbit selection and optimization, as suggested in [7]. This would allow P-MAA to adjust not only for artifact reduction, but also for specific surgical imaging task.

In conclusion, the P-MAA method offers a novel and efficient approach to metal artifact avoidance that is fundamentally different from previous segmentation-based techniques. By leveraging surrogate shape models such as ellipsoids, P-MAA achieves faster and more targeted trajectory optimization, reducing artifacts while maintaining computational efficiency. This work sets the stage for future developments in object-aware optimization and opens the door to more flexible and task-specific metal artifact reduction strategies in clinical imaging.
